# Effects of different sources of potassium fertiliser on yield, fruit quality and nutrient absorption in “*Harward*” kiwifruit (*Actinidia deliciosa*)

**DOI:** 10.1515/biol-2025-1114

**Published:** 2025-07-07

**Authors:** Long Ma, Chunming Chi, Shuangqing Lv, Yan’an Tong, Lili Yang

**Affiliations:** College of Agriculture, Tarim University, Alar, 843300, China; College of Natural Resources and Environment, Northwest A & F University, Yangling, Shaanxi, 712100, China; Research Center of Oasis Agricultural Resources and Environment in Southern Xinjiang, Tarim University, Alar, Xinjiang, 843300, China

**Keywords:** potassium sulphate, potassium chloride, kiwifruit yield, fruit quality, nutrient absorption

## Abstract

Potassium plays an important role in improving crop growth, yield, and quality; however, choosing the right potassium fertiliser remains challenging. To determine the optimal potassium fertiliser for kiwifruit, this study investigated the effects of different potassium sources on kiwifruit yield and postharvest quality as well as plant and soil nutrient contents in an orchard in Shaanxi Province, Northwest China. Two types of potassium fertiliser were examined (K_2_SO_4_ and KCl; total K_2_O = 584 kg ha^−1^) at two different application stages (basal and topdressing) under the following four treatments: basal K_2_SO_4_ + KCl topdressing, basal K_2_SO_4_ + K_2_SO_4_ topdressing, basal KCl + K_2_SO_4_ topdressing, and basal KCl + KCl topdressing. The different potassium sources had no significant effect on kiwifruit yield; however, a slight increase in yield and economic gain was observed under combined treatment with K_2_SO_4_ and KCl compared to single fertiliser treatment. Meanwhile, the single fruit weight and vitamin C content of the fruit were 7.0 and 4.6% higher under treatment with basal K_2_SO_4_ + KCl topdressing compared with K_2_SO_4_ treatment alone, and 3.1 and 14.9% higher compared with KCl treatment alone. Moreover, application of KCl promoted potassium and chlorine absorption by both the leaves and fruit. However, no significant differences in the content of sulphate or chloride ions in the surface soil (0–40 cm) were observed between potassium sources. In contrast, in deeper soil, the content of chloride ions was highest after KCl treatment, while that of sulphate ions was highest after K_2_SO_4_ topdressing. Overall, these findings suggest that the most appropriate potassium source for kiwifruit production is basal application of K_2_SO_4_ followed by KCl as topdressing in the study region.

## 1 Introduction

Potassium is one of the macroelements required for plant growth, playing an important role in a number of physiological processes such as enzyme activation, protein synthesis, photosynthesis, osmoregulation, phloem transport, energy transfer, cation–anion balance, and stress resistance [1,2]. All plants require potassium, especially crops high in carbohydrates such as potatoes and various fruits [3–6]. Crops absorb some potassium from the soil; however, growth and development mainly rely on the application of potassium fertiliser.

Potassium fertilisers include potassium chloride (KCl), potassium sulphate (K_2_SO_4_), potassium dihydrogen phosphate, potassium salt, potassium magnesium salt, carnallite, potassium nitrate, and kiln powder. Of these, KCl and K_2_SO_4_ are most widely used in agricultural production [6–8]. However, due to the different anions involved, the effects of KCl and K_2_SO_4_ on crops are inconsistent [9–11]. A number of studies have examined the effects of different potassium fertilisers on crops [12–14], such as potatoes [15–17], cabbage, cucumber, and lettuce [18–20]; however, little is known about the effects on fruit trees.

Kiwifruit is very popular due to its high nutrient content and desired flavour [21]. China is a major producer of kiwifruit, with production and harvest areas both ranking first in the world [22]. Shaanxi Province is one of the main kiwifruit producing areas in China, which accounts for 32 and 55% of the total planting area and annual production, respectively [23]. Potassium (K) is a crucial plant nutrient, widely recognised as the “quality element” due to its significant role in enhancing the yield and quality of various crops. Potassium deficiency can lead to significant yield losses and quality defects [24]. Kiwifruit has a higher demand for potassium compared to other crops, as potassium is the most abundant mineral element in kiwifruits [25]. With an annual yield reaching tens of thousands of kilograms per hectare, kiwifruit cultivation removes a large amount of potassium from the soil. Therefore, to ensure optimal yield and quality, substantial potassium fertiliser supplementation is required annually in kiwifruit orchards. However, choosing the right potassium fertiliser for kiwifruit production remains a problem [26]. Improper use of chloride-containing fertilisers can have an adverse effect on some sensitive crops [27,28] and, as a result, they are rarely used on cash crops in China. Accordingly, K_2_SO_4_ is therefore the most popular potassium fertiliser; however, kiwifruit tends to prefer K_2_SO_4_, while having a high demand for chlorine [29]. Whether potassium chloride is more suitable than potassium sulphate to increase kiwifruit yield and improve fruit quality?

Overall, the effects of different potassium fertilisers on kiwifruit yield and quality are poorly understood. The objective of this study, therefore, was to examine the effects of two different sources of potassium (K_2_SO_4_ and KCl) applied as different potassium sources and different application method (as basal and topdressing fertilisers) in kiwifruit orchards. The results provide a basis for rational fertiliser management of kiwifruit production.

## 2 Materials and methods

### 2.1 Experimental site

Two experiments were conducted. An experiment was conducted to explore which potash fertiliser is suitable as the potassium source for kiwifruit, called as “Experiment One.” The experimental site was located in Hengqu Town, Meixian County, Shaanxi Province (N34°11′, E107°58′), a semi-humid drought-prone area with an annual average temperature of 12.9°C, annual average of 2015.2 h sunshine, annual average frost-free period of 216 days, and annual average rainfall is 600 mm. The kiwifruit orchard was established in 2006, covering a total area of 0.17 hectares. Tree spacing was 2 × 3 m and the kiwifruit variety planted was “Hayward” (*Actinidia deliciosa*). The rootstock is a seedling, and the scion is “Hayward.” The training system is T-shaped scaffold. The orchard soil was alluvial soil, calcareous soil, with a pH of 8.0, organic matter content of 12.3 g kg^–1^, mineral nitrogen content of 30.8 mg kg^–1^, available phosphorus content of 231.4 mg kg^–1^, and available potassium content of 127.9 mg kg^–1^.

Another experiment is to explore the application methods (as basal or topdressing fertilisers) of different potash fertilisers, called as “Experiment Two.” The experiment site was located in Yangling, Shaanxi Province, China (N34°17′, E108°6′), a semi-humid drought area, with an average temperature of 13.2°C, annual rainfall of approximately 600 mm (mainly concentrated in July to September), and annual evaporation of 1,400 mm. The selected kiwifruit orchard was built in 2005, with a total planted area of 0.2 hectares. *Actinidia deliciosa* “Hayward” scions were grafted onto2-year-old seedlings then planted at a spacing of 1.7 m × 3 m, and growing vines were trained along a T-shaped scaffold. The orchard is flat and comprised Lou soil, calcareous soil, with a pH of 8.2, organic matter content of 11.3 g kg^–1^, mineral nitrogen content of 36.2 mg kg^–1^, available phosphorus content of 222.4 mg kg^–1^, and available potassium content of 154.3 mg kg^–1^.

### 2.2 Experimental design

**Experiment 1.** Three treatments were examined: K_2_SO_4_, potassium sulphate as potash fertiliser; K_2_SO_4_ + KCl, whenever potash fertiliser is applied, it is half potassium sulphate and half potassium chloride; KCl, potassium chloride as potash fertiliser. The experiment was conducted from October 2014 through November 2017. The experimental design employed a randomised block of five chlorine treatments, with three replications per treatment, and seven trees in each plot. Each plot was 42 m^2^. Nitrogen fertilisers were in the form of urea (N 46%), phosphate fertiliser as triple superphosphate (P_2_O_5_ 46%), and potassium fertiliser as potassium sulphate (K_2_O 51%) and potassium chloride (K_2_O 62%). All the treatments maintained consistent N (450 kg ha^−1^), P_2_O_5_ (225 kg ha^−1^), and K_2_O (450 kg ha^−1^) nutrients dose. Fifty percent of all the fertiliser was applied as base fertiliser in autumn, and 50% as topdressing at the young fruit stage.

**Experiment 2.** Four treatments were examined: S + Cl, basal K_2_SO_4_ + KCl topdressing; S + S, basal K_2_SO_4_ + K_2_SO_4_ topdressing; Cl + S, basal KCl + K_2_SO_4_ topdressing; and Cl + Cl, basal KCl + KCl topdressing. To do so, 14 healthy and consistent kiwi plants were selected as a single plot, following a randomised complete block design with three replicates. Under each treatment, the same amount of K_2_O (297 kg ha^−1^) was used for basal and topdressing, while nitrogen and phosphate were maintained at conventional rates (528 kg ha^−1^ N and 363 kg ha^−1^ P_2_O_5_, respectively). Half of all chemical fertilisers were applied as basal fertiliser and the remaining half as topdressing after previous season’s fruit harvest (October 2014) and young fruit stage (June 2015), respectively.

Irrigation, weeding, pruning, and other field management measures were consistent with local farming practices.

### 2.3 Sampling and analysis

At the fruit ripening stage (fruit soluble solids of 6.5%), fruit samples were collected from each of the four directions of growth of the trees in each plot. As a result, approximately 50 samples were collected from each plot. Sampling of leaves was similar to that of the fruit, with samples obtained from the middle of branches bearing fruit, in four directions. The total weight of fruit harvested from each plot was recorded as the production rate, which was then divided by the plot area to determine the yield.

When edible (following storage for 20 days in plastic bags at room temperature), five fruits from each treatment were chopped into a mixed sample to determine the quality indicators. The content of soluble solids was determined using a PAL-1 type sugar concentration detector (ATAGO, Japan). Soluble total sugars were determined by anthrone colorimetry. The titratable acid content was determined via NaOH titration. The vitamin C (Vc) content was determined by 2,6-dichloroindophenol colorimetry. Total nitrogen, phosphorus, and potassium contents of the leaves and fruit were determined using a continuous flow analyser (AA3, Seal Analytical, Germany), an ultraviolet spectrophotometer (UV-1900, Shimazu Co., Ltd, China), and a flame photometer (FP 6410, Shanghai Yidian Analytical Instrument Co., Ltd, China), respectively, after digestion in H_2_O_2_–H_2_SO_4_. The chlorine content was determined using an automatic discontinuous chemical analyser (Cleverchem 200, DeChem-Tech.GmbH, Germany) after dry ashing. The sulphur content was determined by barium sulphate turbidimetry using an ultraviolet spectrophotometer (UV-1900, Shimazu Co., Ltd, China) after digestion in HNO_3_–HClO_4_.

Soil samples were collected near the canopy drip line in fall (after harvest) using a stainless-steel auger. Samples were obtained at 20 cm intervals from a depth of 0–200 cm (Experiment 1) and 0–100 cm (Experiment 2). Three random samples were taken from each plot, mixed into a single composite sample then air-dried at room temperature. After removing the roots and debris, the dry soil samples were ground and passed through a 1 mm sieve then stored in polyethylene bottles. Determination of soil chloride ions (Cl^−^) was carried out based on a soil water ratio (w/v) of 1:5 using an automatic discontinuous chemical analyser (Cleverchem 200, DeChem-Tech. GmbH). Soil available sulphur was determined by barium sulphate turbidimetry using an ultraviolet spectrophotometer (UV-1900, Shimazu Co., Ltd, China).

### 2.4 Data analysis

Statistical analyses were performed using SPSS 20.0 (IBM SPSS, Somers, USA). Experimental data are expressed as mean ± SD. All data were subjected to one-way analysis of variance followed by Duncan’s multiple range test. Differences were considered statistically significant at *P* < 0.05. The relationship between leaf and fruit nutrient contents was examined by Pearson correlation analysis.

## 3 Results

### 3.1 Yield and economic gain

Kiwifruit yield was 48.6, 43.1, 50.5, 45.1 t ha^−1^ under T1 to T4, respectively, with no significant differences among treatments (Table 1). The lowest fertiliser input value was observed under T4, with application of KCl alone. Overall, T3 resulted in the highest yield and net income, followed by T1. These findings suggest that application of both fertilisers is beneficial in terms of kiwifruit yield and economic gain.

**Table 1:** Effects of each treatment on kiwifruit yield and economic gain

Treatment	Yield (t ha^−1^)	Output value (10^4^ RMB ha^−1^)	Input value (10^4^ RMB ha^−1^)	Net income (10^4^ RMB ha^−1^)
T1(S + Cl)	48.6 ± 5.5a	19.46	1.15	18.31
T2(S + S)	43.1 ± 4.6a	17.23	1.31	15.92
T3(Cl + S)	50.5 ± 5.6a	20.21	1.15	19.06
T4(Cl + Cl)	45.1 ± 6.8a	18.40	0.99	17.05

For the yield, different letters in the same column indicate significant difference between treatments (*P* < 0.05). Price of fertilisers: urea, 2.0 RMB kg^−1^; superphosphate, 1.0 RMB kg^−1^; K_2_SO_4_, 6.0 RMB kg^−1^; KCl, 4.0 RMB kg^−1^; and commercial organic fertiliser, 1.0 RMB kg^−1^. In the net income analysis, other costs rather than the cost of the fertiliser were not considered.

### 3.2 Postharvest quality

No significant differences in the contents of soluble solids, soluble sugars, and titratable acid or the sugar/acid ratio were observed between treatments (Table 2). However, T2 with K_2_SO_4_ alone resulted in a single fruit weight of 115.2 g, lower than that under T1 with combined K_2_SO_4_ and KCl application (*P* < 0.05). Meanwhile, T4 with KCl alone resulted in the lowest fruit Vc content of 57.9 mg 100 g^−1^, which was lower than that under combined T1 and T2 treatments (*P* < 0.05).

**Table 2:** Effect of different treatments on kiwifruit postharvest quality

Treatment	Single fruit weight (g)	Vc (mg 100 g^−1^)	Soluble sugar (%)	Titratable acid (%)	Soluble solids (%)	Sugar/acid
T1(S + Cl)	123.3 ± 0.5a	66.5 ± 3.3a	7.4 ± 0.6a	1.28 ± 0.01a	12.1 ± 0.3a	5.8 ± 0.4a
T2(S + S)	115.2 ± 5.2b	63.6 ± 0.2ab	7.8 ± 1.0a	1.28 ± 0.05a	11.9 ± 0.3a	6.1 ± 1.0a
T3(Cl + S)	120.2 ± 3.8ab	59.7 ± 2.2bc	7.8 ± 0.5a	1.34 ± 0.11a	11.7 ± 0.2a	5.9 ± 0.9a
T4(Cl + Cl)	119.6 ± 4.6ab	57.9 ± 1.9c	8.0 ± 0.6a	1.26 ± 0.08a	12.4 ± 0.6a	6.4 ± 0.2a

Different letters in the same column indicate significant differences between treatments (*P* < 0.05).

### 3.3 Nutrient contents of the leaves and fruits

**Figure 1:**
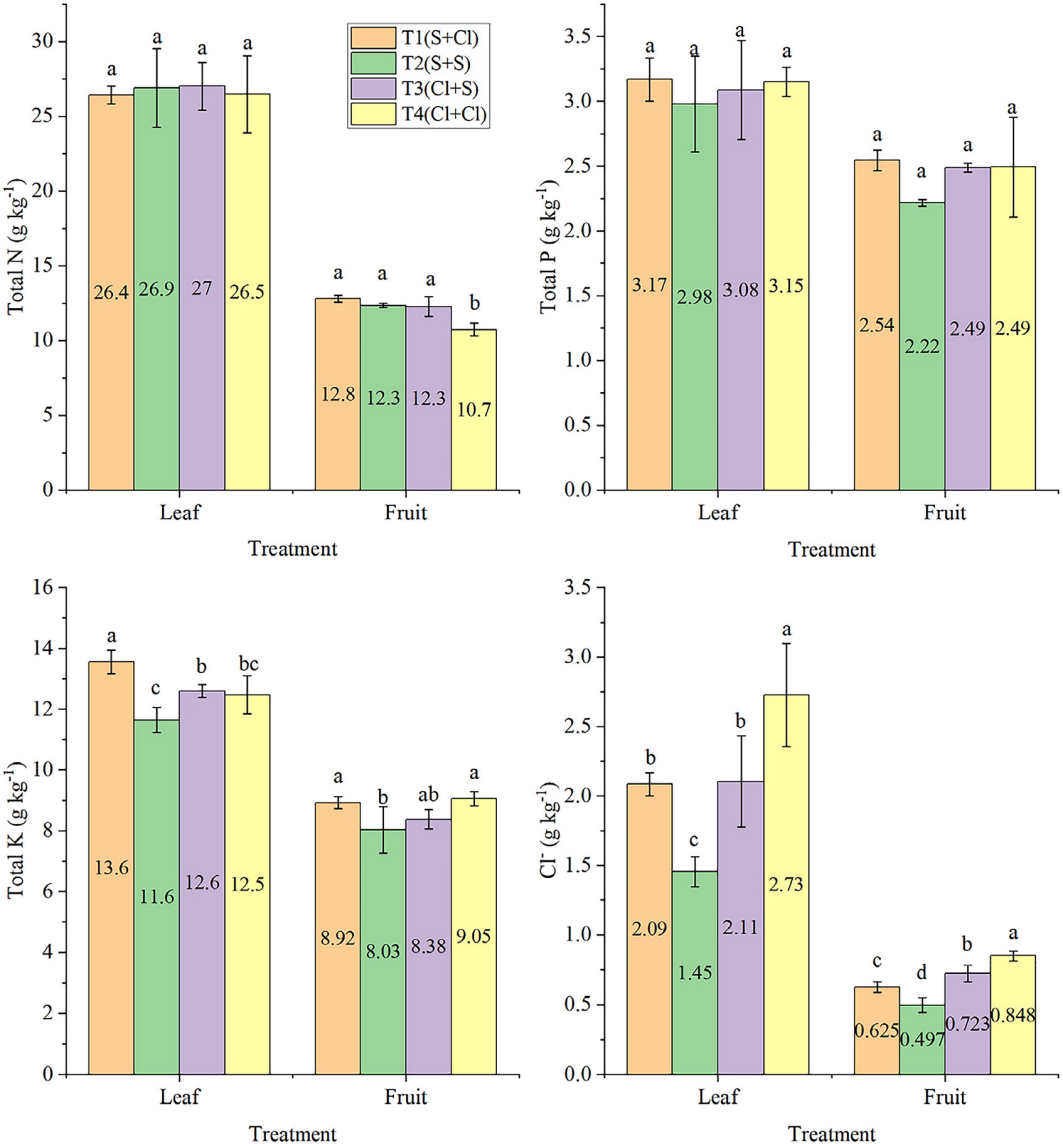
Effects of different treatments on nutrient contents of kiwifruit leaves and fruit at harvest. Different letters above the column represent a significant difference in nutrient content between treatments (*P* < 0.05).

No significant differences in the total nitrogen and phosphorus contents of the leaves were observed between treatments (Figure 1). The leaf potassium content was highest under T1, with K_2_SO_4_ as a basal fertiliser and KCl as topdressing (*P* < 0.05). This was followed by T3, while lowest leaf potassium content was observed under T2. Meanwhile, the fruit potassium content was also higher under T4 with KCl alone compared to T2 with K_2_SO_4_ alone.

The Cl^−^ content of the leaves was positively correlated with the amount of chlorine applied (Figure 1). The leaf Cl^−^ content was highest under T4 and lowest under T2 (*P* < 0.05). The Cl^−^ content of the leaves was about three times that of the fruit, which was also related to the amount of chlorine applied and the application stage. For example, the fruit Cl^−^ content was higher under T4 than T2, while T3 resulted in a higher fruit Cl^−^ content than T1 (*P* < 0.05).

Pearson correlation analysis of the leaf and fruit nutrient contents (Table 3) revealed a significant negative correlation between the leaf nitrogen and potassium contents (*P* < 0.01). Only the leaf and fruit Cl^−^ contents were significantly positively correlated with each other (*P* < 0.05).

**Table 3:** Pearson correlation analysis of leaf and fruit nutrient contents

Leaf N	1							
Leaf P	−0.905**	1						
Leaf K	−0.621	0.86	1					
Leaf Cl	−0.562	0.82	0.429	1				
Fruit N	0.255	−0.249	0.281	−0.733	1			
Fruit P	−0.533	0.946	0.878	0.764	−0.122	1		
Fruit K	−0.877	0.957	0.71	0.869	−0.446	0.821	1	
Fruit Cl	−0.334	0.691	0.287	0.967*	−0.767	0.697	0.719	1
	Leaf N	Leaf P	Leaf K	Leaf Cl	Fruit N	Fruit P	Fruit K	Fruit Cl

*P < 0.05 and **P < 0.01 (2-tailed).

### 3.4 Chloride and sulphate contents of the soil

**Figure 2:**
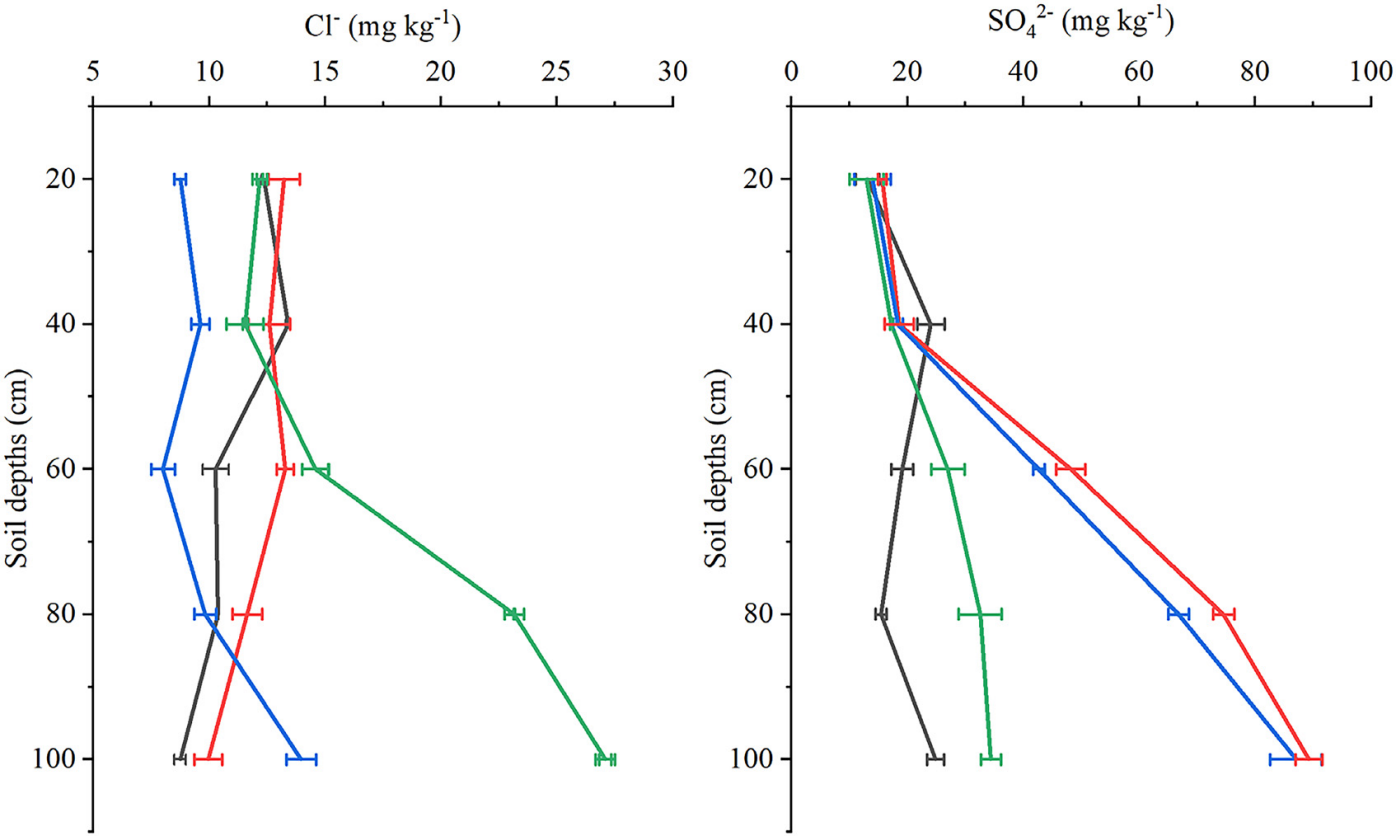
Effects of different treatments on the soil contents of chloride (Cl^−^) and sulphate (SO_4_^2−^) ions at depths of 0–100 cm at kiwifruit harvest.

Very little difference in the Cl^−^ content of the soil was observed between treatment with K_2_SO_4_ alone (T2) and combined treatment with KCl and K_2_SO_4_ (T1 or T3), both of which were below 20 mg kg^−1^ (Figure 2). Meanwhile, the soil Cl^−^ content at depths of 60–100 cm was highest after treatment with KCl alone (T4), with the highest content of 27 mg kg^−1^ at a depth of 80–100 cm. Under all treatments, the SO_4_^2−^ content was below 25 mg kg^−1^ at a soil depth of 0–40 cm, but this increased at depths of 60–100 cm under T1 and T2 compared to T3 and T4. These findings suggest that Cl^−^ and SO_4_^2−^ in the surface soil (0–40 cm) are generally unaffected by potassium fertiliser source, unlike in deeper soil, particularly in the case of SO_4_^2−^. However, by applying K_2_SO_4_ as topdressing, the SO_4_^2−^ content in deeper soil should increase.

## 4 Discussion

The interactions between potassium ions and their companion ions determine the effects of potassium fertiliser on crop growth [30]. The effects of K_2_SO_4_ and KCl remain controversial and have been extensively studied because of their differing anions. While KCl is cheap, it is considered harmful to crop growth and quality compared to K_2_SO_4_. However, these effects vary depending on the crop species, in addition to the amount and stage of fertiliser application [31–33]. Here we examined the effects of two different potassium fertilisers on kiwifruit yield and postharvest quality as well as plant and soil nutrient contents. The results suggest that combined application of K_2_SO_4_ and KCl (T1 and T3) were beneficial in terms of yield, economic gain, postharvest quality, and nutrient contents compared to KCl (T4) and K_2_SO_4_ application alone (T2). This was especially true when K_2_SO_4_ was used as a basal fertiliser and KCl as topdressing for kiwifruit production.

No significant differences in kiwifruit yield were observed between treatments; however, the yield and economic gain were higher under combined K_2_SO_4_ and KCl application. One reason for this is that combined application provides the sulphur and chlorine elements required for kiwifruit growth, both of which function to increase yield. Different crops respond differently to K_2_SO_4_ and KCl under different experimental conditions. For example, application of K_2_SO_4_ and KCl was found to have no significant effect on potato yield, with only a slight increase under KCl treatment [34,35]. However, others suggest that potato yield decreases under KCl compared to K_2_SO_4_ treatment [11,33]. Meanwhile, Inthichack [20] found that celery yield increased under KCl treatment, while cabbage and lettuce yield were higher under K_2_SO_4_ treatment.

The effect of different potassium fertilisers on crop quality is also affected by the amount of potassium applied [31,35], the crop type [36,37], and the experimental conditions [11,16]. In the present study, the effects of different potassium fertilisers on kiwifruit postharvest quality were observed in the single fruit weight and fruit Vc content, both of which were highest under combined application of K_2_SO_4_ and KCl (T1) and lowest under K_2_SO_4_ (T2) and KCl application alone (T4). Meanwhile, other studies suggest that application of chloride-containing fertilisers decreases the Vc content of some crops [38–40]. Although kiwifruit plants require more chlorine than other crops, excess application of KCl can have adverse effects on fruit Vc, possibly due to the physiological metabolism of chlorine [41].

The effect of different potassium fertilisers on crop absorption of nitrogen and potassium varies greatly; however, they have less of an effect on phosphorus absorption [42]. Meanwhile, the absorption of chlorine by crops is positively correlated with the amount of chlorine applied [43,44]. Herein, kiwifruit treated with KCl alone (T4) resulted in the lowest fruit nitrogen content, but the highest fruit Cl and K contents, as well as the highest leaf K and Cl contents. Kiwifruit is well adapted to using Cl^−^ rather than organic-acid anions to charge balance, and therefore, maintain K^+^ absorption [45]. In this study, the K and Cl contents of the kiwifruit leaves and fruit were higher after combined treatment with K_2_SO_4_ and KCl (T1 and T3) compared to K_2_SO_4_ treatment alone (T2), which may be one of the reason for the higher yield and improved quality under T1 and T3. Although the Cl content of the leaves and fruit was highest after treatment with KCl alone (T4), the highest leaf Cl 2.7 g kg^−1^ values did not reach the reported safe concentration for kiwifruit of 20–25 g kg^−1^ [46,47]. It is generally believed that chloride-containing fertilisers inhibit nitrate reductase activity, thereby reducing nitrogen absorption by crops [48]. Moreover, the anion accompanying K^+^ in the solution surrounding plant roots can also influence K^+^ absorption. Notably, SO_4_^2−^ absorption is slow compared to that of other inorganic anions such as CI^−^, which can limit K^+^ absorption [49].

The correlation analysis of nutrients between leaves and fruits can better reflect the relationship between plant nutrients in “source” and “sink.” In this study, their relationship was also analysed to explore whether chlorine in the leaves affects the major mineral nutrients (nitrogen, phosphorus, and potassium) in both leaves and fruits. Different mineral nutrients in plants may exhibit antagonistic or synergistic effects [50]. Some studies suggest a positive correlation between chlorine and nitrogen, phosphorus, and potassium in kiwifruit [25], indicating a synergistic relationship. However, the results of this study are not entirely consistent with those findings, which may be related to differences in kiwifruit varieties and the types of nitrogen fertilisers used.

SO_4_^2−^ and Cl^−^ ions are easily leached into the soil [51], with contents related to soil depth and application rate. Long-term localisation experiments further suggest that SO_4_^2−^ is more likely to accumulate in the soil than Cl^−^ [52]. Moreover, Shen et al. [51] found that long-term application of chloride-containing fertiliser resulted in a consistent soil SO_4_^2−^ content of 26.5 mg kg^−1^, while an increase was observed after application of sulphur-containing fertiliser. In the present study, SO_4_^2−^ and Cl^−^ contents were low in the surface soil (0–40 cm), possibly due to the management of kiwifruit orchards, whereby irrigation is very high at approximately 3,000–6,000 m^3^ ha^–1^ per year. The soil Cl^−^ content remained basically unchanged after treatment with KCl and K_2_SO_4_ alone (T1 and T3); however, in deep soil (80–100 cm) values were highest after treatment with KCl alone (T4, 27 mg kg^−1^; way below the safe concentration of 400 mg kg^−1^ [53]). These findings suggest that normal application of KCl in local kiwifruit orchards does not cause soil or leaf toxicity. Meanwhile, K_2_SO_4_ applied as topdressing (T3 and T2) resulted in an increase in the SO_4_^2−^ content of deep soil, with the highest content of 89 mg kg^−1^. Kiwifruit is sensitive to high levels of SO_4_^2−^ in the soil [46], which may be one of the reasons why the kiwifruit quality was better under T1 than T3.

Considering the fruit yield, postharvest quality, and nutrient contents of the plants and soil, our findings suggest that combined treatment with K_2_SO_4_ as basal fertiliser and KCl as topdressing (T1) is more beneficial for kiwifruit production than treatment with K_2_SO_4_ (T2) or KCl alone (T4). Combined application of K_2_SO_4_ and KCl increased the yield and economic gain while improving fruit quality and increasing the potassium content of both the leaves and fruit. Moreover, it caused no significant increase in salt ions (SO_4_^2−^ and Cl^−^) in the soil. Other studies using different potassium sources also suggest that combined application of K_2_SO_4_ and KCl is superior to K_2_SO_4_ or KCl alone [27]. However, KCl is advocated as a basal fertiliser, to be applied before plant growth in order to allow excessive chlorine to leach from the soil [12,13]. In the present study, using K_2_SO_4_ as a basal fertiliser and KCl as topdressing was the optimal treatment in terms of the various indicators examined. This may be related to the chlorophilic characteristics of kiwifruit. That is, applying KCl as topdressing provides the chlorine required during the growing season, thereby benefiting kiwifruit growth as well as yield and quality.

In conclusion, our findings suggest that combined application of K_2_SO_4_ and KCl in kiwifruit orchards is better than each fertiliser alone. Use of K_2_SO_4_ as a basal fertiliser and KCl as topdressing promotes nutrient absorption, increases yield, and improves postharvest quality of kiwifruit, without causing soil salt toxicity.
